# Autonomic responses in young females with typical vasovagal syncope

**DOI:** 10.1007/s11739-024-03622-7

**Published:** 2024-05-03

**Authors:** Ben Amir, Sapir Barak Lanciano, Ronen Rubinshtein, Udi Nussinovitch

**Affiliations:** 1 Sackler Faculty of Medicine, Tel Aviv University, Tel Aviv, Israel; 2 Department of Cardiology, Edith Wolfson Medical Center, Holon, Israel

**Keywords:** Syncope, Typical vasovagal syncope (tVVS), Heart rate variability (HRV), Autonomic response, Valsalva, Ewing maneuver

## Abstract

To determine whether young women who have experienced typical vasovagal syncope (tVVS) have altered autonomic response parameters, based on a battery of autonomic tests and maneuvers. Notably, previous studies including small cohorts and a partial list of tests yielded conflicting results. A total of 91 otherwise healthy women were included and divided according to those who had experienced tVVS (39 patients) or not (52 patients). Heart rate variability was evaluated at rest, under strict conditions, during 5 min of standing and during a deep breathing test. Response to Valsalva maneuver and Ewing maneuver were also quantified and compared. Both groups had similar clinical characteristics at baseline. No significant differences were found between the two groups in any of the autonomic parameters evaluated. Autonomic responses in young women who experienced typical vasovagal syncope at baseline were indistinguishable from those who did not. Thus, using non-tilt test autonomic screening tests does not seem to provide diagnostic benefits, and may not be useful in predicting recurrence in this patient population.

## Introduction

Syncope is defined as a transient loss of consciousness (TLOC) related to decreased cerebral perfusion. Reasons for syncope are diverse, but the most common cause at a young age is an inadequate autonomic nervous system (ANS) response to specific stimuli. The ANS is comprised of three branches: sympathetic, parasympathetic and enteric [1]. Generally, the sympathetic and parasympathetic systems have opposite effects. For example, a stronger sympathetic signal will increase heart rate, cardiac output and systemic vascular resistance, while the parasympathetic signal will result in a decrease.As a result, inappropriate over-activation of the parasympathetic system and under-activation of the sympathetic system may cause hypotension, followed by TLOC. This chain of events is known as vasovagal syncope (VVS; also known as neuro-cardiogenic syncope) [2]. The true prevalence of syncope is unknown due to the small percentage of patients seeking medical care. Nevertheless, it is estimated that up to 40% of the population will experience TLOC at least once in their lifetime, and that typical VVS (tVVS), as described above, accounts for a substantial majority of syncope cases [3, 4]. This percentage is even higher among women, who have a greater tendency to develop VVS [5]. Additionally, VVS is the most frequent type of syncope in young people, and the relative impact of cardio-inhibition resulting in responsive bradycardia and hypotension declines with age [6, 7]. Although it is mostly considered benign, TLOC might be complicated by physical injury. The high prevalence and potential significance of syncope have led to a wide variety of research. Most studies included a mixed population with varied epidemiological characteristics or populations in which the leading underlying cause may not be tVVS [8–10]. As a result, the literature on the autonomic responses of young females with a predisposition to VVS is limited. In addition, conflicting results have been reported, and most study groups were small, further complicating the interpretation of results [11–13]. The current study endeavored to fill this gap by evaluating the ANS of females ages 30 or younger, in an attempt to identify clinical and physiological parameters associated with a predisposition to tVVS.

## Methods

### Study design

A comparative, cross-sectional study design was used. The local Medical Center Institutional Review Board approved the research protocol. Prior to inclusion, all participants provided written informed consent. Patients with any known medical conditions other than mild dyslipidemia were excluded from the study. Furthermore, any individual taking pharmacotherapy other than oral contraceptives was excluded.

### Participants

Healthy adult female volunteers (< 30 years of age) were recruited from the hospital staff and their family members. The cohort was excluded from inappropriate candidates prior to the conduct of any physiological tests. Patients were questioned about their medical history and 39 reported at least one episode of syncope that was determined to be vasovagal in nature, based on known diagnostic clinical criteria [10]. The control group consisted of 52 seemingly healthy females with no known history of syncope.

### Procedure

Participants were asked to avoid drugs that affect the nervous system and strenuous exercise for 24 h prior to the test, as well as smoking, drinking caffeinated beverages, or taking other stimulants at least 3 h before the study. To prevent sympathetic over-activity, participants were requested to empty their bladder and lie quietly for 10 min before the exam, in a room where the temperature was maintained at 21–23 °C. In all cases, the ECG was conducted between 9:00 a.m. and 12:00 a.m. Blood pressure was measured twice, and the results were averaged. A high-resolution digital ECG with a sampling rate of 2000 Hz (Norav Medical, Yokne’am, Israel) was used for data acquisition. Data collection commenced with the participants lying in a supine position for 15 min. Participants were given 10 min to stabilize their heart rate and avoid distractions. During the last 5 min, recordings of Heart Rate Variability (HRV) parameters and blood pressure were taken. Subsequently, the participants performed a Deep Breathing Test (DBT) and a Valsalva maneuver. Afterward, the participants rested supine for another 5 min. Following that, they were instructed to do an Ewing maneuver, which was followed by a 5 min standing period. Upon completion of this period, the participants were asked to remain standing for an additional 5 min. During that time, HRV parameters were measured, followed with a blood pressure measurement. Orthostatic hypotension was defined as a decrease of 20 mmHg in systolic blood pressure or a decrease in diastolic blood pressure by more than 10 mmHg. Poor quality recordings were repeated. The data were saved and processed using designated commercial computer software (PC-ECG, HRV ver. 5.514, Norav Medical, Yokne’am, Israel) according to accepted standards [14].

### HRV parameters

The mean heart rate (HR) and the square Root of the Mean Squares of the Successive Differences between adjacent NN intervals (RMSSD) were calculated. Additional time-domain parameters were computed by the software, including the standard deviation of RR intervals (SDNN); the absolute normal to normal (NN) intervals that were greater than 50 ms from the preceding ones (NN50) and the ratio of NN50 to the total number of RR intervals recorded (pNN50). The HRV triangular index (HRV-TI) was calculated from an integral of the density distribution (count of all NN intervals) divided by the maximum of the density distribution.

Power spectral analysis was performed using a fast Fourier transform-based nonparametric algorithm. The power spectrum was then converted into frequency-domain indices, which consisted of very-low-frequency (VLF) power (0–0.04 Hz), low-frequency (LF) power (0.04–0.15 Hz), high-frequency (HF) power (0.15–0.4 Hz), and the total power. The latter was computed as the sum of all spectra (i.e., a variance of all NN intervals < 0.4 Hz). HRV indices were log-transformed using the natural logarithm (ln). LF/HF ratio was calculated as an indicator for sympathetic to parasympathetic balance. None of the recordings included premature beats.

### Deep breathing test (DBT)

The participants were asked to remain supine and to breathe deeply in and out six times. Maximum HR during expiration (E) and minimum HR during inspiration (I) were measured, and the difference between the two was calculated (maximum HR – minimum HR = ΔE-I). Values faster than 15 beats per minute are considered within the normal range [16, 17]. The standard deviation of the RR interval and RMSSD were computed for the DB period. The E/I ratio was calculated by dividing the most prolonged RR interval during expiration by the shortest RR interval during inspiration.

### Valsalva maneuver

Valsalva maneuver was performed by having the participants forcefully exhale for 30 s against a closed airway. Initially, the sudden rise in intrathoracic pressure results in decreased afterload and increased preload, thus, producing increased stroke volume. The baroreceptors are then responding to the BP change by increasing the parasympathetic activity, causing responsive bradycardia. However, during a sustained maneuver, the reduced venous return and a drop in arterial pressure cause vagally-mediated tachycardia. Afterward, the peripheral vascular resistance is restored by sympathetic activation. Upon release of the airway obstruction, there is a sudden decrease in intra-thoracic pressure that causes a peak tachycardia, immediately followed by bradycardia until the BP and HR normalize [15]. The ratio between the maximum and minimum measured heart rates during that period was calculated (e.g., Valsalva ratio). Values above 1.2 are considered normal [16]. The tachycardia ratio was calculated by dividing the shortest RR interval during the test by the mean RR interval 30 s before the test. Bradycardia ratio was calculated by dividing the longest RR interval shortly after the test, by the mean RR 30 s before the test. Values of 0.58–1.00 and 0.95–1.60 were considered normal for tachycardia and bradycardia ratios respectively .[17]

### Ewing maneuver

The participants were asked to move from a supine-to-upright position. The sudden active standing causes a decrease in blood pressure and HR elevation after approximately 15 s. After 30 s, blood pressure and HR usually normalize. The ratio between the highest RR interval length after 30 s and the lowest RR interval length after 15 s is referred to as the 15:30 ratio. This parameter reflects the orthostatic cardiac response [16]. Values above 1.04 are usually considered normal [16, 18].

### Statistical analysis

Data were analyzed using JMP version 15 (SAS Institute, Cary, NC, USA). Results are presented as mean and standard deviations. Abnormal results were defined as more than two standard deviations from the normal range. Findings were compared between the groups using Kruskal–Wallis one-way analysis test and Fisher’s Exact Test. P-values less than 0.05 were considered statistically significant.

## Results

The invitation to participate in the study was extended to 108 individuals, of whom 8 declined. Nine more patients were excluded for various reasons (some of whom had more than one criterion making them unsuitable for being labeled as healthy): 7 reported using medications that may affect the autonomic nervous system, 3 had hypothyroidism, 2 had impaired fasting glucose, 5 had a wide range of mood imbalances, and one confessed to past illicit drug use. The remaining 91 healthy young women were recruited, 39 with a history of at least one tVVS event and 52 controls. There were no statistical differences in baseline characteristics between the groups, including age, body mass index, smoking status, family history of ischemic heart disease, family history of diabetes mellitus, hypertension, dyslipidemia, systolic or diastolic blood pressure (Table 1). Five people did not have their blood pressure recorded due to technical reasons or unwillingness to complete the test. Two patients had orthostatic hypotension (2.1%) fitting the epidemiology of that age group [19]. None of the included patients underwent head-up tilt testing (HUTT) due to the high confidence that the event could be classified as typical VVS, or unwillingness to seek medical care. Also, none of them were hospitalized following the tVVS event.

**Table 1:** Demographic characteristics of healthy females who experienced tVVS and controls

Parameter	Syncope (n = 39)	No Syncope (n = 52)	p-value
Age (years)	23.7 ± 3.3	23.7 ± 3.5	0.94
Body mass index (kg/m^2^)	23.6 ± 5.3	22.1 ± 2.4	0.09
Active smoking, n (%)	7 (17.9)	9 (18.4)	0.96
Family history of IHD, n (%)	2 (5.1)	9 (17.3)	0.08
Family history of diabetes mellitus, n (%)	17 (43.6)	20 (38.5)	0.60
Hypertension, n (%)	0 (0)	0 (0)	1.0
Family history of hypertension, n (%)	15 (38.5)	22 (43.1)	0.66
Dyslipidemia*, n (%)	4 (10.3)	3 (5.7)	0.43
SBP at rest (mmHg)**	112.4 ± 8.6	112.0 ± 7.8	0.84
DBP at rest (mmHg)**	74.0 ± 6.2	71.9 ± 7.0	0.13
SBP after 5 min standing (mmHg)**	112.0 ± 7.9	111.8 ± 9.2	0.93
DBP after 5 min standing (mmHg)**	76.6 ± 8.2	73.7 ± 9.5	0.06

*IHD*  ischemic heart disease, *SBP* Systolic blood pressure, *DBP* Diastolic blood pressure, *mild dyslipidemia treated with only lifestyle modification

^**^Blood pressure values were recorded for 86 participants (Syncope group, n = 39; No syncope group, n = 47)

DBT results were excluded for 2 participants due to electrical interference that produced unreliable recordings. Also, standing HRV recordings of 29 participants were excluded due to interference related to artifacts. Forty participants could not perform the Valsalva maneuver correctly. Ewing’s maneuver results were excluded for 13 participants due to faulty recordings. Technical interferences and inadequate maneuvering affected both groups. The results of the adequately performed autonomic tests were collected, analyzed and compared (Tables 2, 3, 4, 5, 6). Analysis of all results showed no significant differences in the parameters tested.

**Table 2:** Basal 5-minues HRV analysis after 10 min of supine rest in tVVS and controls

Parameter	Syncope (n = 39)	No Syncope (n = 52)	p-value
Maximal RR (ms)	1041.2 ± 164.5	1109.9 ± 212.6	0.10
Minimal RR (ms)	670.1 ± 98.0	672.0 ± 105.4	0.93
Average RR (ms)	860.8 ± 118.6	886.8 ± 130.4	0.33
SDNN (ms)	61.1 ± 28.0	71.4 ± 34.5	0.13
RMSSD (ms)	61.2 ± 49.6	69.8 ± 40.8	0.37
HRV triangular index	17.6 ± 6.1	18.6 ± 7.2	0.47
NN50 (n)	45.3 ± 34.7	55.4 ± 35.4	0.18
pNN50 (%)	13.9 ± 11.4	16.7 ± 11.4	0.24
VLF (ms^2^)	176.8 ± 83.3	155.9 ± 84.3	0.24
LF (ms^2^)	155.8 ± 78.4	165.1 ± 66.4	0.55
HF (ms^2^)	200.6 ± 93.5	211.4 ± 88.0	0.57
Total power (ms^2^)	584.9 ± 99.1	575.7 ± 85.2	0.64
LF/HF	1.2 ± 1.4	1.0 ± 0.7	0.47
ln (NN50)	3.3 ± 1.4	3.6 ± 1.3	0.27
ln (pNN50)	2.1 ± 1.3	2.3 ± 1.4	0.37
ln (VLF)	5.0 ± 0.6	4.9 ± 0.6	0.31
ln (LF)	4.9 ± 0.6	5.0 ± 0.5	0.34
ln (HF)	5.2 ± 0.5	5.3 ± 0.5	0.42
ln (total power)	6.4 ± 0.3	6.3 ± 0.2	0.95
ln (LF/HF)	– 0.3 ± 0.9	– 0.3 ± 0.8	0.89

*RR*  time between two successive R-waves, *HR*  heart rate, *SDNN*  standard deviation of NN interval, *RMSSD*  root mean square of successive differences, *HRV*  heart rate variability, *NN50*  number of times successive heartbeat intervals exceed 50ms, *VLF*  very low frequency, *LF*  low frequency, *HF*  high frequency

**Table 3:** HRV analysis with deep breathing test in patients with tVVS and controls

Parameter	Syncope (n = 38)	No Syncope (n = 51)	p-value
Minimum inspiratory HR (bpm)	56.3 ± 8.8	56.1 ± 8.6	0.92
Maximum expiratory HR (bpm)	95.5 ± 8.9	99.3 ± 21.6	0.31
SDNN (ms)	115.8 ± 37.5	121.5 ± 41.1	0.50
ΔE/I	39.2 ± 9.1	43.1 ± 19.7	0.25
E/I ratio	1.7 ± 0.3	1.8 ± 0.3	0.36
RMSSD (ms)	88.4 ± 43.5	89.8 ± 49.9	0.89
VLF (ms^2^)	72.3 ± 57.6	93.9 ± 88.2	0.19
LF (ms^2^)	413.7 ± 87.6	397.6 ± 100.5	0.43
HF (ms^2^)	116.3 ± 56.3	121.5 ± 66.2	0.70
Total power (ms^2^)	609.3 ± 65.9	621.6 ± 71.3	0.41

*RR*  time between two successive R-waves, *HR*  heart rate, *SDNN*  standard deviation of NN interval, ΔE/I = Maximum heart rate during expiration subtracted by minimum heart rate during inspiration, *E/I ratio*  Ratio of maximum and minimum heart rate during expiration and inspiration, *RMSSD*  root mean square of successive differences, *VLF*  very low frequency, *LF*  low frequency, *HF*  high frequency

**Table 4:** HRV analysis recorded in a standing position in patients with tVVS and controls

Parameter	Syncope (n = 27)	No Syncope (n = 35)	p-value
Max RR (ms)	847.1 ± 91.8	889.9 ± 194.2	0.30
Min RR (ms)	533.3 ± 128.9	574.7 ± 112.7	0.18
Average RR (ms)	686.9 ± 89.2	713.3 ± 143.4	0.41
SDNN (ms)	48.4 ± 8.9	51.7 ± 23.8	0.51
RMSSD (ms)	28.7 ± 16.0	31.8 ± 30.5	0.63
HRV triangular index	14.5 ± 2.6	15.1 ± 4.4	0.48
NN50	15.5 ± 19.7	15.6 ± 21.9	0.98
pNN50	1.3 ± 1.6	1.6 ± 2.5	0.58
VLF (ms^2^)	299.5 ± 105.1	290.8 ± 104.9	0.75
LF (ms^2^)	217.1 ± 86.8	233.8 ± 86.1	0.45
HF (ms^2^)	82.2 ± 52.2	104.2 ± 70.0	0.18
Total power (ms^2^)	753.9 ± 156.6	743.5 ± 146.4	0.79

*RR*  time between two successive R-waves, *HR*  heart rate, *SDNN*  standard deviation of NN interval, *RMSSD*  root mean square of successive differences, *HRV*  Heart Rate Variability. *NN50*  number of times successive heartbeat intervals exceed 50ms. *VLF*  very low frequency, *LF*  low frequency, *HF*  high frequency

**Table 5:** Valsalva analysis in patients with tVVS and controls

Parameter	Syncope (n = 21)	No syncope (n = 30)	p-value
RR minimum phase 2 (ms)	710.7 ± 98.4	719.7 ± 125.0	0.78
RR maximum phase 4 (ms)	936 ± 166.2	959.5 ± 151.2	0.60
Valsalva ratio	1.3 ± 0.1	1.4 ± 0.2	0.53
Tachycardia ratio	0.8 ± 0.2	0.8 ± 0.1	0.45
Bradycardia ratio	1.0 ± 0.3	1.1 ± 0.2	0.26

*RR*  time between two successive R-waves

**Table 6:** Ewing maneuver analysis in patients with tVVS and controls

Parameter	Syncope (n = 34)	No syncope (n = 44)	p-value
RR minimum 15s standing (ms)	566.4 ± 80.6	563.6 ± 77.5	0.88
RR maximum 30s standing (ms)	834.4 ± 113.7	860.5 ± 113.0	0.34
30:15 ratio	1.5 ± 0.2	1.6 ± 0.3	0.26

*RR*  time between two successive R-waves

## Discussion

Little is known about the relation between HRV and syncope. Although a few reports studied the hypothesized relationship, the results seem to be contradictory and conclusions indecisive. The PRISE pilot study found no significant differences in HRV values when examining risk groups for syncopal events in the general population ages 16 or older[10]. It should be noted that the study included a small number of participants, making the results less conclusive. Additionally, Lagro et al. studied a geriatric population and failed to find a correlation [12]. In contrast, Piccirillo et al. found that HRV analysis differs in patients with recurrent syncope and in whom HUTT induces syncope [13]. Similarly, Miranda et al. found that spectral analysis can identify patients with syncope and cardioinhibitory response in the general population ages 14 or older [20]. In a younger population, Stewart et al. found differences in HRV in adolescents ages 12–17 while comparing healthy and syncope groups [21]. Others suggested that the inability of HRV to predict future TLOC events in patients with cardioinhibitory and neurocardiogenic syncope [22, 23]. Thus, HRV has been shown to be an inadequate marker for syncopal events in this clinical setting by some reports, but other autonomic testing has been inadequately studied. In line with some previous reports, in the current study, HRV analysis showed that parameters strongly related to VVS, most noticeably the sympathetic parameters (LF, LF/HF, VLF), were not statistically different between the two groups. This suggests that analysis of baseline sympathetic tone is not a reliable means of determining a tendency towards development of syncopal events in younger women. HRV analysis during DBT also showed no statistical significance, as opposed to past observations [13]. Thus, the previous findings and assumptions that HRV spectral analysis parameters are suitable to establish a tendency for syncope may not apply to young women [13, 20, 21].

Moreover, all the above suggest that HR variations at baseline and following various autonomic stimuli are also not sufficient to determine a tendency for syncopal events in young women. Furthermore, the study results showed no statistical differences in the Valsalva ratio (Table 5) or the 15:30 ratio between groups (Table 6). Nevertheless, we cannot rule out the possibility of its utility in other populations. We could not find substantial reporting of Valsalva or the 30:15 ratio for assessing a tendency for syncope in young female individuals in the literature. More extensive research is required to determine the true potential of these autonomic tests.

This study had a few limitations. Only women who agreed to participate join the cohort. Therefore, there is a potential for selection bias. Due to the lack of data regarding the timing of the most recent syncope event, a potential confound can arise, since autonomic responses may fluctuate over time. Additionally, we used participants’ anamnesis for the study, which may have been affected by factors that could affect memory. Due to the subjects’ young age and lack of substantial medical workup, it is likely reasonable to estimate the results represent those who experienced tVVS a few months or years prior to inclusion, and that relapse was not common. Recollection bias is further limited in this age group as abnormalities in memory function are less likely. There are other possible technical limitations that might have affected the results. The Valsalva and the 15:30 ratios are performance-dependent. Consequently, despite guidance from the researcher, there was a high number of exclusions that potentially reduced the generalizability and statistical power of the study. It remains unknown if the results would have been different if exclusion of tests due to technical issues was less common. Yet, both study and control group were affected and hence the exclusion of test by itself would have only minor effect on inter-group comparisons. In addition, we did not use the HUTT in this study. Although it is still in use in those presenting with typical VVS or suspected reflex syncope (a IIa indication for HUTT), it usually adds little to patient management. Also, there are some concerns regarding its sensitivity, specificity, yield, complications and availability for wide-use screening, mainly due to its limited diagnostic value in cases of syncope due to an uncertain cause. Moreover, about 11–14% of healthy controls are found to have abnormal tilt results, thus making the interpretation of results more challenging [24].

## Conclusions

The results of this study indicate that HRV spectral analysis parameters during standing and DBT, Valsalva ratio and 15:30 ratio analysis did not find differences between young women with or without a history of tVVS. Thus, the study emphasizes the difficulty of distinguishing between people at risk for VVS and those who are not. Further research is needed in order to determine the sensitivity of these tests to predict syncope in other patient groups and in recurrent TLOC, and determine if the autonomic tests changes over the course of time since the primary event.

## Data Availability

Data will be provided upon a reasonable request.
